# Evaluating the Optimal Sequence of Treatment With EGFR Inhibitors and Bevacizumab in RAS Wild-Type Metastatic Colorectal Cancer

**DOI:** 10.7759/cureus.23543

**Published:** 2022-03-27

**Authors:** Diana Martins, Jéssica Rodrigues, Patrícia Redondo, Ivo Julião, Cátia Faustino

**Affiliations:** 1 Medical Oncology, Instituto Português de Oncologia do Porto Francisco Gentil, EPE, Porto, PRT; 2 Cancer Epidemiology Group, IPO Porto Research Center of IPO Porto (CI-IPOP)RISE@CI-IPOP (Health Research Network), Portuguese Oncology Institute of Porto (IPO Porto)Porto Comprehensive Cancer Center (Porto.CCC), Porto, PRT; 3 Management, Outcomes Research and Economics in Healthcare Group (MOREHealth) Research Center of IPO Porto (CI-IPOP), Portuguese Institute of Oncology Francisco Gentil of Porto, Outcomes Research Lab, Porto, PRT

**Keywords:** oncology, sequence, egfr inhibitors, bevacizumab, metastatic colorectal cancer

## Abstract

Background

Epithelial growth factor receptor inhibitors (EGFRi) and bevacizumab are the two main target therapies available for first-line treatment of RAS wild-type (wt) metastatic colorectal cancer (mCRC). However, the optimal sequencing of these agents remains unclear. In this study, we aimed to evaluate the optimal sequence with EGFRi and bevacizumab in first- and second-line treatment.

Methods

This was a retrospective cohort study with RAS wt mCRC patients identified by extended RAS analysis between 2013 and 2020 at a comprehensive cancer center. All patients had to be treated with a sequence of systemic treatment that included an EGFRi and bevacizumab in first and second line, in either order. Two groups were defined according to treatment sequence: first-line EGFRi followed by second-line bevacizumab (cohort A) or the reverse sequence (cohort B). Primary endpoint was overall survival (OS). Secondary endpoints were progression-free survival with first-line treatment (PFS1), progression-free survival with second-line treatment (PFS2), objective response rate (ORR), and serious adverse events (grade ≥ 3). Survival was estimated using the Kaplan-Meier method, and survival differences between groups were compared using the log-rank test. Univariate analyses were performed using Cox proportional hazard model.

Results

A total of 124 patients were included (93 in cohort A and 31 in cohort B). There were no statistical significant differences in median OS (A: 34.9 months vs B: 29.2 months; p=0.590), PFS1 (A: 13.1 months vs B: 8.2 months; p=0.600), and PFS2 (A: 7.4 months vs B: 5.5 months; p=0.110) between groups. No significant differences were also found between treatment sequences in subgroups defined by age, gender, primary tumor location, sidedness, timing of metastasis, number of metastatic sites, multimodal therapy, primary tumor resection, and first-line chemotherapy backbone. ORR was significantly higher with first-line treatment with EGFRi (A: 55.9% vs B: 22.6%; p=0.001). At the final follow-up, the proportion of patients with SAEs was similar between treatment sequences (p=0.827).

Discussion

Our study showed no impact of the treatment sequence with EGFRi and bevacizumab in the survival of RAS wt mCRC. However, patients treated with first-line EGFRi had significantly higher response rates, thus favoring its use in patients with symptomatic tumors and borderline resectable metastasis. Prospective trials are warranted to define the optimal sequence of treatment in RAS wt mCRC patients.

## Introduction

According to the most recent data from GLOBOCAN, colorectal cancer (CRC) is the third most common cancer and the second leading cause of cancer-related death worldwide [1]. In 2020, there were more than 1.93 million new CRC cases and 935,000 CRC-related deaths in the world [1]. Approximately 25% of the patients with CRC present with metastatic disease at diagnosis and 40-50% will develop metastasis during follow-up [2]. With the development of multimodal therapies and increasing use of targeted agents, median overall survival (mOS) of metastatic colorectal cancer (mCRC) has improved to more than 30 months in selected studies [3].

Current molecular targeted therapies, available for first-line treatment, include bevacizumab, which targets the vascular endothelial growth factor (VEGF), and cetuximab or panitumumab, which targets the epithelial growth factor receptor (EGFR) [4]. While there are no validated predictive molecular biomarkers of response to bevacizumab, RAS mutations (exons 2-4 of KRAS and NRAS) represent a negative predictive marker for anti-EGFR therapy, and, consequently, its use is limited to RAS wild-type (wt) population [5-7].

The optimal target agent for first-line treatment of RAS wt patients has not been established [3,8]. The FIRE-3 and CALGB/SWOG 80405 trials compared the efficacy of first-line chemotherapy with either cetuximab or bevacizumab but provided conflicting results [3,8]. The first trial did not meet its primary endpoint of objective response rate (ORR) but showed an overall survival (OS) benefit for cetuximab, contrasting with the second one that showed similar progression-free survival (PFS) and OS in both treatment groups [3,8]. A possible explanation may be the impact of subsequent later‐line therapies and the sequence of targeted therapies [9]. Retrospective analysis have suggested that the prior use of bevacizumab may impair the effect of cetuximab or panitumumab, thus favoring EGFR inhibitors (EGFRi) as the optimal choice for first-line treatment [9-12]. However, this was not uniformly replicated in other studies [13,14].

In this study, we aimed to evaluate the optimal sequence of treatment with EGFRi and bevacizumab in patients with RAS wt mCRC.

## Materials and methods

Study design and participants

This was a retrospective cohort study that included all consecutive RAS wt mCRC patients submitted to extended RAS analysis between January 2013 and December 2020 at a comprehensive cancer center in Portugal (Portuguese Oncology Institute of Porto). Eligibility criteria were as follows: (i) histologically confirmed diagnosis of CRC; (ii) confirmed KRAS (exons 2- 4) and NRAS (exons 2-4) wt genotype; (iii) sequential first-line treatment with an EGFRi (cetuximab or panitumumab) followed by second-line bevacizumab, or the reverse sequence, in combination with chemotherapy; and (iv) documented disease progression between first and second line. Patients who had undergone less than two cycles of targeted therapy were excluded. Demographic, clinical, treatment, effectiveness, and toxicity data were abstracted from clinical and administrative records.

The study was approved by the Institutional Ethics Committee. Due to its retrospective nature, the requirement for informed consent was waived for this study.

Study outcomes

The primary endpoint was OS, defined as the time from the start of first-line treatment to death or last follow-up. Secondary endpoints were progression-free survival 1 (PFS1), defined as the time from initiation of first-line treatment to disease progression on first line or death; progression-free survival 2 (PFS2), defined as the time from initiation of second-line treatment to disease progression on second line or death; and objective response rate (ORR), defined as the proportion of patients with complete or partial response as best response according to RECIST (Response Evaluation Criteria in Solid Tumours) criteria (version 1.1). Safety was evaluated by the proportion of patients with grade ≥ 3 adverse events (AEs) according to the Common Terminology Criteria for Adverse Events (CTCAE). The cut-off date for the analysis was August 31, 2021.

Statistical analysis

Patients were categorized into two cohorts according to sequence of treatment received: first-line EGFRi followed by bevacizumab (cohort A) and first-line bevacizumab followed EGFRi (cohort B). Categorical variables were summarized as frequencies and percentages. Continuous variables were presented as median, minimum, and maximum. Comparison between groups of continuous variables was performed using the Mann-Whitney U test, and the association of categorical variables and ORR was assessed using the chi-square test or the Fisher’s exact test. Survival analysis was performed using the Kaplan-Meier method and survival differences were compared with log-rank test. Univariable Cox regression analysis was used to calculate differences in survival for selected subgroups. A p-value of less than 0.05 was considered statistically significant. Data analysis was performed using the R software v4.0.5 (R Foundation for Statistical Computing, Vienna, Austria).

## Results

Patient characteristics and treatment

**Figure 1:**
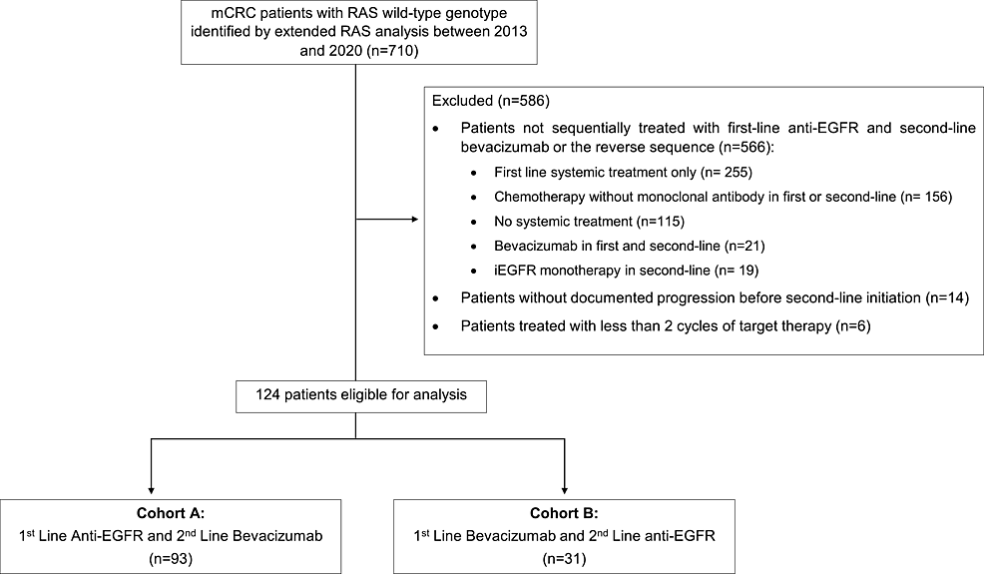
Consolidated Standards of Reporting Trials (CONSORT) diagram depicting patient selection. mCRC, metastatic colorectal cancer; EFGR, epithelial growth factor receptor

In total, 710 medical records of patients with RAS wt mCRC were reviewed. After applying all the inclusion and excluding criteria, 124 patients were included for the analysis: 93 patients (75.0%) received a first-line anti-EGFR followed by second-line bevacizumab (cohort A) and 31 patients (25.0%) received the reverse treatment sequence (cohort B) (Figure 1).

Patients and disease characteristics are summarized in Table 1. Median age at diagnosis was 57.7 years (24.0-74.0). Most patients were female in cohort A (66.7%) and male in cohort B (54.8%) (p=0.055). Compared with cohort B, cohort A had a significantly higher proportion of patients with synchronous metastatic disease (A: 61.3 % vs B: 38.7%; p=0.047). In addition, more patients in cohort A had metastasis limited to the liver (48.4% vs 16.1%; p=0.003). Both groups had mainly left-sided tumors (82.8% vs 80.6%; p=0.790), a non-mucinous histology (97.8% vs 90.3%; p=0.099), and low grade of differentiation (46.2% vs 45.2%; p=0.464). No significant imbalances were detected for the proportion of BRAF-V600E mutations (7.5% vs 19.4%; p=0.091) and microsatellite instability (5.4% vs 12.9%; p=0.345).

**Table 1:** Baseline patient and tumor characteristics in overall population: cohort A and cohort B. ^a^Right-sided colon: cecum, ascending colon, hepatic flexure, or transverse colon; ^b^Left-sided colon: splenic flexure, descending colon, sigmoid colon or rectum. ^c^Grade of histologic differentiation according to the WHO classification. ^d^Number of organs involved by metastasis. ^e^Other sites: metastasis located at other sites than lung, liver, peritoneum, or non-regional lymph nodes. EGFR, epithelial growth factor receptor; ECOG PS, Eastern Cooperative Oncology Group performance status; NOS, not otherwise specified; CEA, carcinoembryonic antigen

Baseline patient characteristics	Overall (n=124)	Cohort A (anti-EGFR/bevacizumab) (n=93)	Cohort B (bevacizumab/anti-EGFR) (n=31)	p-Value
Age at diagnosis, median (range), years	57.5 (28.0-74.0)	57.0 (30.0-74.0)	59.0 (28.0-74.0)	0.876
Gender				
Male	48 (38.7%)	31 (33.3%)	17 (54.8%)	0.055
Female	76 (61.3%)	62 (66.7%)	14 (45.2%)	
ECOG PS				
0-1	124 (100.0%)	93 (100.0%)	31 (100.0%)	-
Primary tumor location				
Colon	75 (60.5%)	56 (60.2%)	19 (61.3%)	1.000
Rectum	49 (39.5%)	37 (39.8%)	12 (38.7%)	
Tumor sidedness				
Right sided^a^	22 (17.7%)	16 (17.2%)	6 (19.4%)	0.790
Left sided^b^	102 (82.3%)	77 (82.8%)	25 (80.6%)	
Histology				
Adenocarcinoma, NOS	119 (96.0%)	91 (97.8%)	28 (90.3%)	0.099
Mucinous adenocarcinoma	5 (4.0%)	2 (2.2%)	3 (9.7%)	
Grade^c^				
Low grade	57 (46.0%)	43 (46.2%)	14 (45.2%)	0.464
High grade	11 (8.9%)	7 (7.5%)	4 (12.9%)	
Unknown	56 (45.2%)	43 (46.2%)	13 (41.9%)	
Microsatellite instability				
No	73 (58.9%)	57 (61.3%)	16 (51.6%)	0.345
Yes	9 (7.3%)	5 (5.4%)	4 (12.9%)	
Unknown	42 (33.9%)	31 (33.3%)	11 (35.5%)	
BRAF status				
Wild-type	53 (42.7%)	44 (47.3%)	9 (29.0%)	0.091
Mutated	13 (10.5%)	7 (7.5%)	6 (19.4%)	
Unknown	58 (46.8%)	42 (45.2%)	16 (51.6%)	
Timing of metastasis				
Metachronous	55 (44.4%)	36 (38.7%)	19 (61.3%)	0.047
Synchronous	69 (55.6%)	57 (61.3%)	12 (38.7%)	
Number of metastatic sites^d^				
1	80 (64.5%)	63 (67.7%)	17 (54.8%)	0.279
>1	44 (35.5%)	30 (32.3%)	14 (45.2%)	
Metastatic sites				
Liver only	50 (40.3%)	45 (48.4%)	5 (16.1%)	0.003
Lung only	11 (8.9%)	7 (7.5%)	4 (12.9%)	
Liver and other sites	28 (22.6%)	22 (23.7%)	6 (19.4%)	
Peritoneal only	6 (4.8%)	2 (2.2%)	4 (12.9%)	
Non-regional lymph nodes only	11 (8.9%)	7 (7.5%)	4 (12.9%)	
Others^e^	18 (14.5%)	10 (10.8%)	8 (25.8%)	
CEA, median (range), ng/mL	14.8 (1.2-17572.0)	25.9 (1.2-17572.0)	9.8 (1.4-1558.0)	0.063

Treatment exposure is described in Table 2. Patients in cohort B were more likely to be previously treated with adjuvant chemotherapy (29.0% vs 67.7%; p<0.001) and pelvic irradiation (17.2%vs 41.9%; p=0.010). A higher proportion of patients in cohort A had irinotecan-based regimens (FOLFIRI or irinotecan) as first-line chemotherapy (93.5% vs 51.6%; p <0.001). Patients in cohort A were more likely to receive oxaliplatin-based regimens (FOLFOX or CAPOX) as second-line chemotherapy (90.3% vs 19.4%; p<0.001). Multimodal therapies (metastasis resection and/or ablative therapies) were performed in 38.7% in cohort A and 35.5% in cohort B (p=0.748). Metastasectomy was conducted in 31.2% patients in cohort A and 19.4% in cohort B (p=0.205). In the subset of patients with synchronous metastasis, primary tumor resection was conducted in 38 (40.9%) patients of cohort A and 9 (29.0%) of cohort B (p=0.739).

**Table 2:** Treatment exposure in overall population: cohort A and cohort B. ^a^Resection of metastasis or ablative therapies (hepatic thermoablation, chemoembolization, radioembolization, hepatic/pulmonary SBRT) EGFR, epithelial growth factor receptor; SBRT, stereotactic body radiation therapy

Treatment exposure	Overall (n=124)	Cohort A (anti-EGFR/bevacizumab) (n=93)	Cohort B (bevacizumab/anti-EGFR) (n=31)	p-Value
First-line chemotherapy backbone				
Oxaliplatin-based	21 (16.9%)	6 (6.5%)	15 (48.4%)	<0.001
Irinotecan-based	103 (83.1%)	87 (93.5%)	16 (51.6%)	
Second-line chemotherapy backbone				
Oxaliplatin-based	90 (72.6%)	84 (90.3%)	6 (19.4%)	<0.001
Irinotecan-based	29 (23.4%)	6 (6.5%)	23 (74.2%)	
Others	5 (4.0%)	3 (3.2%)	2 (6.5%)	
Multimodal therapy^a^				
No	77 (62.1%)	57 (61.3%)	20 (64.5%)	0.748
Yes	47 (37.9%)	36 (38.7%)	11 (35.5%)	
Metastasis resection	35 (28.2%)	29 (31.2%)	6 (19.4%)	0.205
Primary tumor resection				
No	22 (17.7%)	19 (20.4%)	3 (9.7%)	0.278
Yes	102 (82.3%)	74 (79.6%)	28 (90.3%)	
In patients with synchronous metastasis	47 (37.9%)	38 (40.9%)	9 (29.0%)	0.739
Prior adjuvant chemotherapy				
No	76 (61.3%)	66 (71.0%)	10 (32.3%)	<0.001
Yes	48 (38.7%)	27 (29.0%)	21 (67.7%)	
Prior pelvic irradiation				
No	95 (76.6%)	77 (82.8%)	18 (58.1%)	0.010
Yes	29 (23.4%)	16 (17.2%)	13 (41.9%)	

Survival outcomes

**Figure 2:**
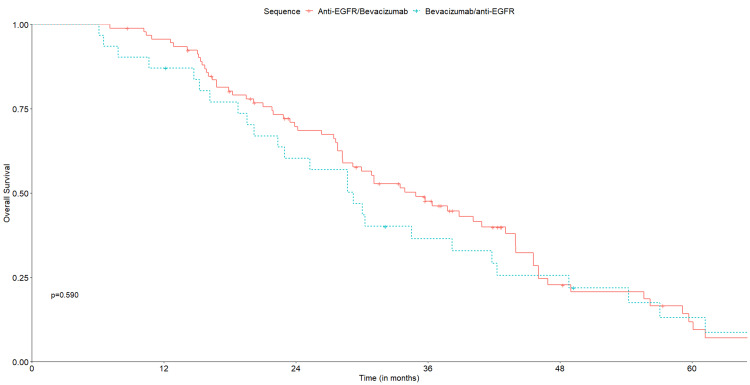
Kaplan‐Meier curves for overall survival of the two treatment sequences.

**Figure 3:**
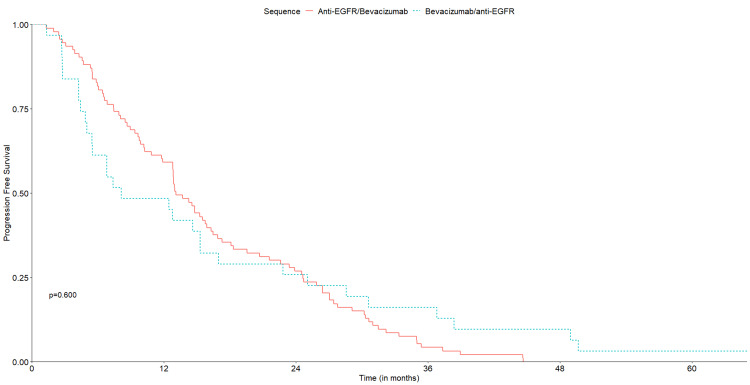
Kaplan‐Meier curves for PFS1 of the two treatment sequences. PFS1, progression-free survival with first-line treatment

**Figure 4:**
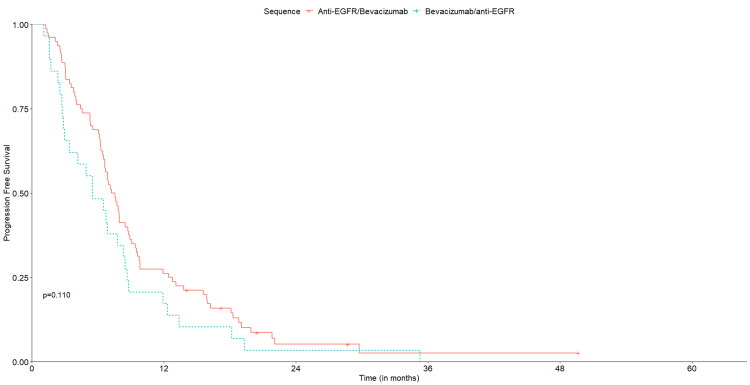
Kaplan‐Meier curves for PFS2 of the two treatment sequences. PFS2, progression-free survival with second-line treatment

Median follow-up was 29.4 months for cohort A (95% CI: 6.1-87.3) and 30.0 months for cohort B (95% CI: 6.1-80.6). OS was similar between treatment groups, with an mOS of 34.9 months in cohort A (95% CI: 28.3-44.0) and 29.2 months (95% CI: 22.4-42.3) in cohort B (p=0.590) (Figure 2). No significant differences were observed for median PFS1, with 13.1 months (95% CI: 11.9-16.3) in cohort A and 8.2 months (95% CI: 5.5-22.8) in cohort B (p=0.600) (Figure 3). Median PFS2 was also similar, with 7.4 months (95% CI: 6.7-88) in cohort A and 5.5 months (95% CI: 3.4-8.5) in cohort B (p= 0.110) (Figure 4).

No significant survival differences were observed between treatment sequences in subgroup analyses conducted according to age, gender, primary tumor location, sidedness, timing of metastasis, number of metastatic sites, multimodal therapy, primary tumor resection, and first-line chemotherapy backbone (Table 3).

**Table 3:** Univariable Cox-proportional hazards model for OS, PFS1, and PFS2 OS, overall survival; EGFR, epithelial growth factor receptor

Variables	OS			PFS1			PFS2		
	HR	95% CI	p-Value	HR	95% CI	p-Value	HR	95% CI	p-Value
Age									
<58 years									
Anti-EGFR/Bevacizumab	1.00			1.00			1.00		
Bevacizumab/anti-EGFR	0.90	0.47-1.74	0.758	0.87	0.45-1.69	0.683	0.63	0.32-1.26	0.192
≥58 years									
Anti-EGFR/Bevacizumab	1.00			1.00			1.00		
Bevacizumab/anti-EGFR	1.39	0.73-2.64	0.312	1.50	0.82-2.74	0.184	1.49	0.83-2.65	0.178
Gender									
Male									
Anti-EGFR/bevacizumab	1.00			1.00			1.00		
Bevacizumab/anti-EGFR	1.38	0.73-2.59	0.325	1.69	0.91-3.16	0.096	1.16	0.64-2.13	0.623
Female									
Anti-EGFR/bevacizumab	1.00			1.00			1.00		
Bevacizumab/anti-EGFR	1.10	0.55-2.20	0.777	1.03	0.54-1.95	0.936	0.77	0.41-1.46	0.418
Primary tumor location									
Colon									
Anti-EGFR/bevacizumab	1.00			1.00			1.00		
Bevacizumab/anti-EGFR	1.01	0.56-1.83	0.968	1.55	0.89-2.69	0.121	0.94	0.55-1.61	0.811
Rectum									
Anti-EGFR/bevacizumab	1.00			1.00			1.00		
Bevacizumab/anti-EGFR	1.36	0.64-2.92	0.445	0.67	0.32-1.40	0.284	0.89	0.44-1.83	0.759
Tumor sidedness									
Left-sided									
Anti-EGFR/bevacizumab	1.00			1.00			1.00		
Bevacizumab/anti-EGFR	1.09	0.65-1.83	0.743	1.06	0.65-1.72	0.828	0.80	0.49-1.31	0.378
Right-sided									
Anti-EGFR/bevacizumab	1.00			1.00			1.00		
Bevacizumab/anti-EGFR	0.97	0.36-2.59	0.949	1.78	0.66-4.76	0.254	1.77	0.67-4.69	0.253
Timing of metastasis									
Synchronous									
Anti-EGFR/bevacizumab	1.00			1.00			1.00		
Bevacizumab/anti-EGFR	1.06	0.51-2.19	0.873	1.64	0.82-3.26	0.161	0.84	0.42-1.68	0.626
Metachronous									
Anti-EGFR/bevacizumab	1.00			1.00			1.00		
Bevacizumab/anti-EGFR	1.18	0.63-2.21	0.613	0.92	0.50-1.68	0.776	1.00	0.56-1.79	0.994
Number of metastatic sites									
1									
Anti-EGFR/bevacizumab	1.00			1.00			1.00		
Bevacizumab/Anti-EGFR	1.13	0.63-2.03	0.682	0.99	0.57-1.72	0.976	0.95	0.55-1.66	0.867
>1									
Anti-EGFR/bevacizumab	1.00			1.00			1.00		
Bevacizumab/anti-EGFR	1.27	0.60-2.68	0.530	1.30	0.62-2.71	0.488	0.96	0.47-1.95	0.901
Multimodal therapies									
Anti-EGFR/bevacizumab	1.00			1.00			1.00		
Bevacizumab/anti-EGFR	1.61	0.74-3.52	0.230	1.55	0.75-3.20	0.238	1.33	0.67-2.65	0.408
Primary tumor resection									
Anti-EGFR/bevacizumab	1.00			1.00			1.00		
Bevacizumab/anti-EGFR	1.10	0.67-1.80	0.704	1.08	0.68-1.73	0.732	0.86	0.54-1.37	0.534
First-line chemotherapy									
Oxaliplatin									
Anti-EGFR/bevacizumab	1.00			1.00			1.00		
Bevacizumab/anti-EGFR	0.68	0.18-2.58	0.571	0.52	0.18-1.56	0.244	0.73	0.26-2.05	0.549
Irinotecan									
Anti-EGFR/bevacizumab	1.00			1.00			1.00		
Bevacizumab/anti-EGFR	1.41	0.80-2.50	0.233	1.62	0.92-2.84	0.094	0.90	0.50-1.60	0.717

Response rates

Response rates are summarized in Table 4. Patients treated with a first-line EGFRi in cohort A had a significantly higher ORR with first-line treatment (55.9%) than patients treated with first-line bevacizumab in cohort B (22.6%) (p=0.001). In cohort A, 53.7% of patients had partial responses (vs 19.4% in cohort B) and 3.2% patients had complete responses (vs 3.2% in cohort B).

**Table 4:** Response rates to first-line treatment in cohort A (EGFRi) and cohort B (bevacizumab) ^a^ORR with first-line treatment ORR, overall response rate; EGFRi, epithelial growth factor receptor inhibitors

		Cohort A (n=93)	Cohort B (n=31)	p-Value
Best response, n (%)	Complete response	3 (3.2)	1 (3.2)	
	Partial response	49 (52.7)	6 (19.4)	
	Stable disease	32 (34.4)	16 (51.6)	
	Progressive disease	9 (9.7)	8 (25.8)	
ORR^a^, %		55.9	22.6	0.001

Safety

At the final follow-up, there were no significant differences in the rate of SAEs (grade ≥ 3) between treatment groups (Table 5). The rate of suspension of monoclonal antibody due to toxicity was low for both groups (8.6% vs 6.5%) with no statistically significant differences.

**Table 5:** Serious AEs (≥ grade 3) in cohort A and cohort B ^a^Number of patients with at least one grade ≥ 3 AE AE, adverse event; VEGF, vascular endothelial growth factor; GI, gastrointestinal; EGFR, epithelial growth factor receptor

	Cohort A (anti-EGFR/bevacizumab), n=93	Cohort B (bevacizumab/anti-EGFR), n=31	p Value
	No. (%)	No. (%)	
Grade ≥ 3 AEs^a^	61 (65.6)	21 (67.7)	0.827
VEGF inhibitor-specific			
Hypertension	6 (6.5)	1 (3.2)	0.679
Proteinuria	1 (1.1)	2 (6.5)	0.154
Hemorrhage	3 (3.2)	0 (0)	0.572
Thrombosis	1 (1.1)	0 (0)	1.000
GI perforation	0 (0)	0 (0)	-
EGFR inhibitors class-specific			
Cutaneous	19 (20.4)	3 (9.7)	0.175
Infusional reaction	2 (2.2)	0 (0)	1.000
Hematologic	42 (45.2)	15 (48.4)	0.755
Non-hematologic			
GI	4 (4.3)	2 (6.5)	0.639
Neurological	3 (3.2)	1 (3.2)	1.000
Hepatic	1 (1.1)	0 (0)	1.000
Pulmonary	0 (0)	0 (0)	-
Others	2 (2.2)	2 (6.5)	0.260
Antibody suspension due to toxicity	8 (8.6)	2 (6.5)	1.000
EGFR inhibitor	2 (2.2)	0 (0)	1.000
Bevacizumab	6 (6.5)	2 (6.5)	1.000

## Discussion

We conducted a real-world retrospective study that included RAS wt mCRC patients sequentially treated with EGFRi and bevacizumab in the largest comprehensive cancer in Portugal with a long follow-up period. This study is of particular interest since data regarding the best treatment sequence with these therapies is lacking and, also because, most studies that compared bevacizumab and EGFRi in first-line setting did not account for the impact of subsequent therapies [3,8].

In our study, we found no significant survival differences between mCRC patients treated with a first-line anti-EGFR followed by second-line bevacizumab versus the reverse treatment sequence. Additionally, there were no significant differences regarding the time to treatment failure in either first or second lines. In contrast, we observed a significantly higher ORR in patients treated with an EGFRi during first-line treatment.

The findings in our study are consistent with the published data from the CALGB 80405 trial [3]. While this trial did not show significant differences in OS and PFS, response rates were significantly higher with first-line cetuximab [12]. Our study reinforces the data that EGFRi may be a more suitable option when cytoreduction is the main goal of treatment, especially in patients with symptomatic tumors and borderline resectable metastasis needing tumor response [15].

Our study, however, contrasts with the data from the FIRE-3 trial [8]. A retrospective analysis of this study showed that patients treated with bevacizumab in second-line after previous first-line cetuximab had a longer second-line survival. This study suggested that first-line treatment with an EGFRi followed by second-line bevacizumab may be a more suitable option than reverse sequence [9]. Additionally, a recent meta-analysis of 13 randomized trials and four observational studies evaluating the efficacy of different target therapy sequences showed an OS benefit for cetuximab followed by bevacizumab, in comparison with the reverse order, regardless of the chemotherapy backbone [16]. In vitro studies have supported this data as they have shown that EGFRi resistance was associated with a higher sensitivity to anti-VEGF therapy, while previous treatment with bevacizumab impaired the sensitivity to EGFRi through an EGFR-independent RAS activation [17-19].

It should be noted, nevertheless, that some prospective clinical trials found no detrimental impact of prior VEGF inhibition on the efficacy of subsequent therapies. The SPIRITT randomized phase II trial assessed the efficacy of second-line treatment with FOLFIRI plus panitumumab or bevacizumab, after progression on FOLFOX plus bevacizumab, and reported no significant differences in survival between both target therapies [20]. Similarly, the randomized ASPECCT trial that compared cetuximab and panitumumab in chemotherapy-refractory patients reported similar survival for those who had been previously treated with bevacizumab versus those who had no prior bevacizumab treatment [21]. These data suggest that for patients willing to avoid the cutaneous toxicity of EGFRi the choice of bevacizumab may also be an adequate option with no detriment in long-term outcomes.

Recently, it has been proposed that tumor sidedness may help in the selection of the optimal first-line target agent [22]. A retrospective analysis of FIRE-3 trial has shown that patients with left-sided tumors had a longer second-line survival with a sequence of first-line cetuximab and second-line anti-VEGF, as compared with the reverse sequence. This benefit was, however, not observed in patients with right-sided tumors [23]. In our study, tumor sidedness was not associated with a differential benefit according to treatment sequence. However, these data must be interpreted with caution as there may have been a potential bias due to the low number of patients with right-sided tumors.

The results of our study provided further real-world evidence on the use of EGFRi and bevacizumab in mCRC and, due to the fact that all patients have been exposed to both types of treatment, we were able to evaluate the potential interaction between them. However, our study had several limitations. These were inherent to its retrospective nature, the limited small sample size in subgroups, and the bias regarding treatment selection, which depended on the physician’s choice. Further validation with prospective multicenter studies is required. Ongoing clinical phase III trials (CR-SEQUENCE and STRATEGIC-1) are evaluating the sequential approach of these targeted therapies in mCRC and may in the future elucidate on the optimal sequencing of these agents [24,25]. Until they yield their results, treatment selection should be determined by patients’ clinical features and preferences, tumor characteristics, toxicities, and goal of treatment.

## Conclusions

In conclusion, while there remains considerable ongoing debate regarding the optimal use and sequence of biologic therapy in mCRC, our data support the evidence that EGFRi may be a more suitable first-line option as it provides higher tumor-response rates. Our data do not support, however, the hypothesis that first-line bevacizumab use negatively impacts the subsequent efficacy of anti-EGFR therapy. Further prospective research is needed to clarify the present results.
